# Acceptability and Usability of HCV Self-Testing in High Risk Populations in Vietnam

**DOI:** 10.3390/diagnostics11020377

**Published:** 2021-02-23

**Authors:** Linh Thuy Nguyen, Van Thi Thuy Nguyen, Kim Anh Le Ai, Minh Binh Truong, Tam Thi Minh Tran, Muhammad S. Jamil, Cheryl Johnson, Elena Ivanova Reipold, Philippa Easterbrook, Kidong Park

**Affiliations:** 1 Center for Bio-Medical Research, National Institute of Hygiene and Epidemiology, Hanoi 100000, Vietnam; linh.nguyen.2@ucdconnect.ie (L.T.N.); tamtranskku@gmail.com (T.T.M.T.); 2 Country Office in Viet Nam, World Health Organization, Hanoi 100000, Vietnam; parkk@who.int; 3 Thai Nguyen Provincial Centre for Disease Control, Thai Nguyen 24000, Vietnam; laka0280@gmail.com (K.A.L.A.); bsminhcdtdltn@gmail.com (M.B.T.); 4 Global HIV, Hepatitis and STI Programmes, World Health Organization, 1202 Geneva, Switzerland; mjamil@who.int (M.S.J.); johnsonc@who.int (C.J.); easterbrookp@who.int (P.E.); 5 Foundation for Innovative New Diagnostics, 1202 Geneva, Switzerland; Elena.Ivanova@finddx.org

**Keywords:** HCV, self-testing, oral fluid, PWID, MSM, Vietnam

## Abstract

HIV self-testing has emerged as a safe and effective approach to increase the access to and uptake of HIV testing and treatment, especially for key populations. Applying self-testing to hepatitis C virus (HCV) may also offer an additional way to address low coverage of HCV testing and to accelerate elimination efforts. To understand the potential for HCV self-testing (HCVST), an observational study was conducted to assess the acceptability and usability of the OraQuick^®^ HCV Self-Test (prototype) among people who inject drugs (PWID) and men who have sex with men (MSM) in Thai Nguyen, a province in northern Vietnam. A total of 105 PWID and 104 MSM were eligible and agreed to participate in the study. Acceptability, defined as the proportion of participants among eligible subjects who agreed to participate in the study, was 92.9% in PWID and 98.6% in MSM. Compared to MSM, PWID were older (median age: 45 vs. 22 years; *p* < 0.0001) and had a lower education level (high school and college: 38.1% vs. 100%; *p* < 0.0001). HCVST usability was high among MSM with fewer observed mistakes, difficulties, or participants requiring assistance (33.7%, 28.8%, and 17.3%, respectively) compared to PWID (62.9%, 53.3%, and 66.7%, respectively; all *p* < 0.0001)). Inter-reader and inter-operator agreement were good in both groups (Kappa coefficient range: 0.61–0.99). However, the concordance between HCVST and study staff -read or performed HCV testing was lower among PWID than MSM (inter-reader concordance 88.6% vs. 99.0% and inter-operator concordance 81.9% vs. 99%). Overall, HCVST was highly acceptable with moderate to high usability among PWID and MSM in Thai Nguyen. Efforts to provide support and assistance may be needed to optimize performance, particularly for PWID populations and for those who are older and with lower literacy or education levels.

## 1. Introduction

Viral hepatitis B and C are major causes of morbidity and mortality worldwide. Together, they lead to approximately 1.34 million deaths per year [1]. In 2015, an estimated 71 million people had chronic hepatitis C virus (HCV) infection worldwide, and around three-quarters of them lived in low- and middle-income countries [1]. Despite the availability of treatment and a cure for HCV, less than 20% of people with chronic hepatitis C have been tested and are aware of their infection [1].

Most transmission of HCV in high-income settings, including North America and Western and Eastern Europe, is due to previous or current injecting drug use. In all regions, injecting drug users are at higher risk of acquiring HCV than the general population, and two-thirds of people who inject drugs (PWID) are estimated to have been infected with HCV [2]. A high prevalence of HCV infection among men who have sex with men (MSM) was recently reported in Taiwan with the HCV incidence per 1000 person years increased from 12.9 in 2014 to 25.4 in 2018 [3]. In this study, syphilis infection was associated with HCV seroconversion, which suggested that behaviors such as unprotected anal sex and chemsex can increase the risk of sexually transmitted infections including HCV. Unsafe injection and medical practices also contribute significantly to new infections in many low- and middle-income countries with generalized epidemics. The most affected regions are Eastern Mediterranean region and north and west Africa, where most infections are caused by unsafe medical injections and other medical procedures.

Vietnam is among the 20 countries with the highest burden of chronic HCV infection in the world and PWID are the most affected population. HCV antibody prevalence among the general population in Vietnam ranged from 0.2–3.3% [4], but it is substantially higher among PWID (74–87%) [5], HIV-infected individuals (22.9–89.0%) [6], HIV-infected MSM (83.1–100%) and HIV-uninfected MSM (8.9–28.2%) [7], and multi-transfused and dialysis patients (6–26.6%) [8,9]. A modelling exercise conducted by the Vietnam Ministry of Health and World Health Organization (WHO) in 2017 estimated that there were nearly one million people with chronic HCV infection in Vietnam. Although no disaggregated data are available on HCV infection among high-prevalence key populations, the most affected groups are PWID, MSM, and people who have had chronic hemodialysis and multiple blood transfusions. The national response to viral hepatitis is based on a national action plan on prevention and control of viral hepatitis (2015–2019) that presents a set of comprehensive measures for prevention, alongside testing and treatment. Since 2019, direct-acting antivirals (DAA) have been included in the health insurance reimbursement list. However, the patient copayment rate is still as high as 50%, resulting in limited access to HCV treatment. Advocacy for the reduction of patient copayment to 20% is ongoing.

The recent introduction of DAAs has transformed treatment options for persons with chronic hepatitis C infection, leading to cure in more than 90% of treated individuals [10,11]. The WHO Global Health Sector Strategy on Viral Hepatitis 2016–2021 has a goal to eliminate viral hepatitis C and B infection as a public health threat by 2030—defined as a reduction in mortality by 65% and new infections by 90% [2]. The attainment of this goal will require 90% of those infected to be diagnosed, and 80% of those diagnosed to be treated. Therefore, the substantial scale-up of testing and treatment of chronic HCV infection will be required from the current level, where less than 20% of those infected are diagnosed.

WHO recommends treatment for all HCV-infected persons with pan-genotypic regimens regardless of stage of the disease. WHO also recommends focused testing in the most affected populations. These include those who have a history of exposure or high-risk behaviors for HCV infection (e.g., PWID, people in prisons and other closed settings, MSM, sex workers, HIV-infected persons, and children of mothers with chronic HCV infection especially if co-infected with HIV), as well the general population in settings with an HCV antibody prevalence ≥2–5% [12]. Although testing can be performed using simple and affordable rapid diagnostic tests (RDT), access to HCV testing services and confirmatory HCV viral load testing remains a major barrier to scale-up of treatment and elimination of the disease. Many of those affected in high HCV burden countries live in rural or remote settings or are members of high-risk marginalized populations such as PWID or MSM that are hard to reach and stigmatized. Consequently, the uptake of HCV testing and treatment in these high-risk populations remains low, and new strategic approaches to increase HCV testing coverage are needed.

WHO recommends a range of HCV testing approaches, including a combination of simple and affordable RDTs and confirmatory HCV viral load testing, particularly for those most affected by chronic HCV infection [12]. Access to these services, however, is limited in many settings due to stigma and discrimination, particularly for PWID and MSM, who also have high HCV prevalence and incidence. New strategic approaches to increase HCV testing coverage especially in key populations such as PWIDs and MSM are needed.

One such approach is self-testing. In self-testing, an individual performs a simple rapid test and interprets their own result, and it has been shown to significantly increase the uptake of HIV testing services [13,14]. Numerous studies have shown self-testing to be safe, accurate, effective and acceptable to many populations unreached by existing services [13,15,16,17]. Since 2016, WHO has recommended self-testing for HIV and currently lists four WHO prequalified products for self-testing [18,19]. As of July 2020, 88 countries had national policies supporting self-testing and 41 were fully implementing—including Vietnam where HIVST is widely implemented among key populations [20].

As for HIV, self-testing for HCV has potential to increase access to HCV testing for those unreached by existing services and to contribute to efforts to treat, cure, and eliminate HCV by 2030. To adapt and introduce self-testing for HCV, it is important to understand the usability and acceptability among potential users. Thus, to inform the development of HCV self-testing (HCVST) policy at global, regional, and national levels, we report on findings from an observational study exploring HCVST acceptability and usability among PWID and MSM in Vietnam.

## 2. Objective

The main study objectives were: (1) to determine the acceptability of and preferences for HCVST among populations at high-risk for HCV infection (PWID and MSM) and (2) to determine the usability (the ability to correctly perform test and interpret results) of an HCV self-test.

## 3. Material and Methods

### 3.1. Study Design and Sample Size

This was an observational study. As there was no published data on acceptance and usability of HCV self-testing, we made a conservative assumption that 50% of eligible individuals will accept self-testing. To estimate the proportion in this study with a 95% confidence interval based on Wilson’s score method, with ±10% margin of error, a minimum sample size of 100 participants was required. 

It was expected that the two groups would screen approximately 200 eligible subjects during the recruitment period and approximately 100 participants would agree to perform the test. In fact, we were able to reach higher number of eligible participants with higher response rate. A total of 209 participants (105 PWID and 104 MSM) were included in the study compared to the target of 100.

### 3.2. Setting and Participant Recruitment

The study was conducted in Thai Nguyen province, North Vietnam. Thai Nguyen is one of the provinces with a high burden of HIV, and it also has strong networks of PWID and MSM which have led community-based HIV testing since 2017 [20].

Peer educators recruited participants by reaching out to PWID and MSM communities and using a short questionnaire to identify eligible individuals. The recruitment took place at the offices of the three community-based organizations (two for PWID and one for MSM) where community-based- and key population-led HIV testing is provided. In addition, an online approach was also applied through social media (Facebook, Zalo, etc.).

Those eligible were invited to participate in the study, with separate sessions organized for PWID and MSM, and offered an OraQuick HCV self-test kit (OraSure Technologies Inc., Bethlehem, PA, USA). Those who agreed to participate provided a written informed consent prior to study enrollment. Individuals with reactive results were referred to further testing and treatment at a nearby facility according to the national guidance.

### 3.3. Study Population—Eligibility Criteria

Individuals who self-identified as PWID and/or MSM were considered eligible and enrolled in the study if they were ≥18 years old, were able to provide written informed consent, had negative or unknown HCV status in the past 12 months, were able to read Vietnamese, and had no previous experience self-testing for HIV and/or HCV. PWID enrolled also had to report use of un-prescribed intravenous drugs within the past 12 months. MSM enrolled also had to report at least one anal sex episode with another man within the past 12 months.

### 3.4. Study Procedures

Baseline information on demographic characteristics, exposure to risk factors, and previous experience with HCV testing services was collected via questionnaires. The study participants were provided with written and pictorial instructions in Vietnamese on how to perform the HCVST and then were asked to perform the test and interpret the result. The HCVST kit contained: one OraQuick^®^ HCV Rapid Antibody Test (OraSure Technologies Inc., Bethlehem, PA, USA), developer solution, test stand (plastic), desiccant, and manufacturer-provided instructions for use. While the OraQuick^®^ HCV Rapid Antibody Test is prequalified by WHO for professional use, all participants were informed that the kit and instructions for self-testing were for research purposes only.

HCVST was performed at community-based organization offices—where community-based testing for HIV is regularly conducted. In these key population-led testing sites, 5 principles of HIV testing including privacy were applied. Same principles were applied for HCVST.

A study staff member silently observed the participants and documented any error in testing procedure using a standardized checklist. The study staff did not provide any assistance unless specifically requested by a participant or after multiple efforts to conduct the test unassisted.

After self-testing was completed and the participants read their results, the test result was re-read by a study staff member to measure inter-reader concordance. Then, a brief post-testing interview was conducted to collect acceptability and preferences. Following this, to measure inter-operator concordance, we compared the self-tester’s results to a second OraQuick^®^ HCV Rapid Antibody Test that was performed by a study staff member who was blinded to the self-reported results.

Post-test counselling was provided by study staff from provincial Centre for Disease Control. The key messages provided to individuals with reactive results were the meaning of HCVST positive results, the necessity of retesting in health facility, the benefit of HCV treatment, and the location of the health facility where participants could access HCV testing, diagnosis, and treatment in Thai Nguyen province.

### 3.5. Data Analysis

The results are reported as percentage and median (range) as appropriate. The statistical significance of differences between groups was analyzed by the Fisher’s exact test and Chi square test, and *p* values less than 0.05 were considered statistically significant. Usability was assessed by calculating the frequencies of mistakes, difficulties and assistance needed at each step of the testing procedure from opening the package to reading the results. Inter-reader concordance for self-test results was defined as agreement between the results interpreted by the participant and by a study staff member, reported as a percentage. Inter-operator concordance was defined as agreement between the results of the self-testing reported by the participant and results of professional use test conducted by a study staff member, reported as a percentage. Cohen’s Kappa statistic was calculated for both inter-reader and inter-operator concordance. When there was only one rating, Gwet’s AC coefficient was used to assess level of agreement. Acceptability and attitudes about self-testing were reported as frequencies and described descriptively. We also used Chi-square test to explore the factors associated with user errors. The statistical analysis was performed with software MedCalc version 14.8.1 (MedCalc Software Ltd, Acacialaan, Ostend, Belgium).

### 3.6. Ethical Approval

The Review Board in Bio-medical Research at National Institute of Hygiene and Epidemiology, Hanoi, Vietnam approved the study on 30 July 2019 (IRB-VN01057/IORG 0008555, number NIHE IRB—20/2019).

## 4. Results

### 4.1. Patient Recruitment and Characteristics

**Figure 1:**
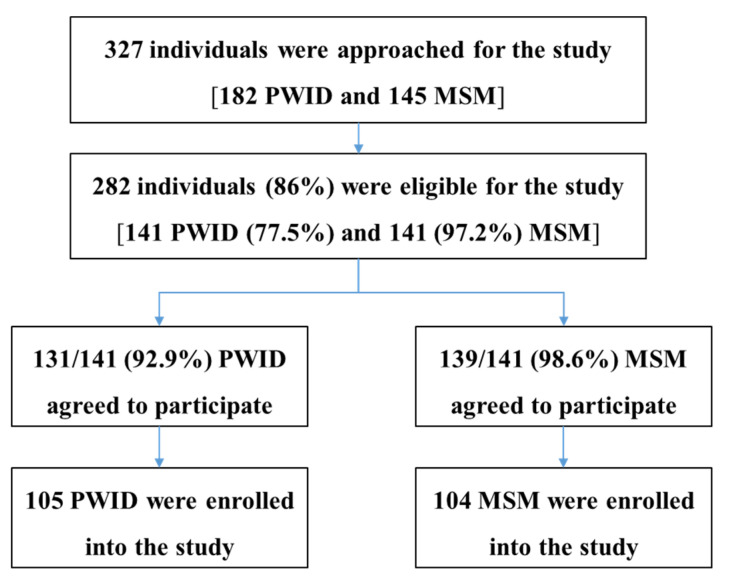
Flowchart of eligible individuals who were enrolled in the study (PWID: people who inject drugs and MSM: men who have sex with men).

A total of 327 individuals (182 PWID and 145 MSM) were approached and screened for eligibility. Among 282 eligible participants (141 PWID and 141 MSM), 270 (95.7%) agreed to participate in the study (131 PWID and 139 MSM). However, due to the time delay between agreement and enrollment, only 77.4% (209/270) participants were available and enrolled in the study (105 PWID and 104 MSM) during the data collection period, October–December 2019, (Figure 1).

The baseline characteristics of participants in the two groups are shown in Table 1. Compared to the MSM group, PWID were more likely to be older (median age: 45 vs. 22 years; *p* < 0.0001), less educated (college, the highest level obtained in both groups: 1% in PWID vs. 87.5% in MSM, *p* < 0.0001) and more unemployed (48.6% in PWID vs. 2.9% in MSM; *p* < 0.0001). MSM were also more likely to be aware of self-testing than PWID (87.5% vs. 32.4%; *p* < 0.0001). Among two cohorts, only one participant in PWID had experience with self-testing, which was related to monitoring glucose as part of diabetes management.

### 4.2. Usability of Self-Testing Performed by Participants

HCVST procedures included completing 12 steps starting with opening the pouch and ending with interpreting the test result. Table 2 reports observer assessment of user mistakes completing steps overall and Table 3 reports observed difficulties and required assistance while self-testing.

Both groups were generally able to self-test without errors. PWID were more likely to incorrectly interpret their HCVST result compared to MSM (20% vs. 1%; *p* < 0.0001) (Table 2). The most common mistake observed was incorrect collection of the oral fluid specimen (37.1% in PWID and 13.5% in MSM; *p* = 0.0001). The second most common mistake was touching the flat pad (used for collecting oral fluid) (6.7% in PWID and 3.8% in MSM; *p* = 0.538).

For those needing assistance, the most difficult step was removing the cap from the developer solution (PWID: 21% of PWID and MSM: 19.2%) (Table 3). Sliding the developer solution into the stand was more challenging for PWID than MSM (11.4% vs. 1.9%; *p* = 0.010). PWID needing assistance also had more difficulty interpreting their result correctly than did MSM (13.3% vs. 1.9%; *p* = 0.003).

PWID also required more assistance, at all steps, than MSM (Table 2 and Table 3). Among all participants with at least one mistake observed, PWID with and without study staff assistance made significantly more errors than MSM with or without study staff assistance. Any observed mistake, difficulty and provided assistance in the PWID group (62.9% [66/105], 53.3% [56/105] and 66.7% [70/105], respectively) were significantly higher than in the MSM group (33.7% [35/104], 28.8% [30/104] and 17.3% [18/104], respectively). The difference in proportions of any mistake, difficulty, and assistance between the two groups were statistically significant.

We also analyzed the association between demographic characteristics and levels of usability (mistakes, difficulties or provision of assistance). We found older age (>45 years), lower educational level (primary or intermediate school), and marital status (married) were associated with higher proportion making either a mistake, having trouble or requiring assistance (Table 4).

### 4.3. HCV Testing Results

**Figure 2:**
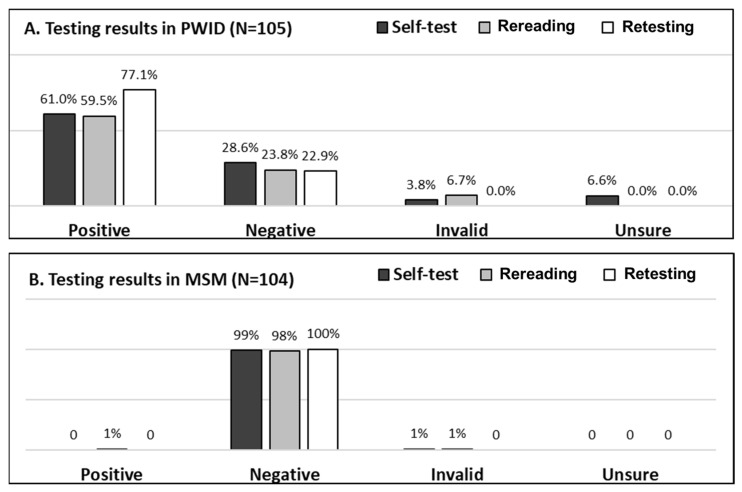
Summary of HCV testing results in PWID (**A**) and MSM (**B**) for participant self-testing, rereading by trained staff, and retesting performed by trained staff.

The HCV testing results performed by the participants (self-testing), re-interpreted by the study staff (rereading), and performed by study staff (retesting) in PWID and MSM are summarized in Figure 2. The self-testing results of the PWID group were 61.0% positive, 28.6% negative, 3.8% invalid, and 6.6% unable to interpret. Compared to the reference result determined with retesting performed by study staff, the positive rate with self-testing was lower in PWID group (61% vs. 77.1%; *p* = 0.01). In MSM group, 99% participants interpreted their results as negative and 1% as invalid while on rereading by study staff, one was read as a positive result. However, on retesting, the result was confirmed negative.

Table 5 shows the concordance of results between those interpreted by participants and study staff in both groups. In the PWID group, the Cohen’s Kappa coefficient for inter-reader agreement was 0.77. The concordance of inter-reader results in this group was 88.6% (93/105). There were five cases in which the participants interpreted the results as negative, but the results were all positive when reread by study staff. In another seven cases, where the participants were not able to interpret their results, the study staff reread and found four cases had positive results and three cases had invalid results. For inter-operator agreement in PWID, the Cohen’s Kappa coefficient was 0.61. The concordance of results between “self-testing” and “retesting” was 81.9% (86/105). There were eight cases in which the participants interpreted the results as negative, but they were all positive when the study staff retested. In the other four cases, which the participants interpreted as invalid results, two cases were positive and two were negative on retesting. There were seven cases in which the participants were not able to interpret their results, and all the results were positive when the seven participants were retested by staff.

In the MSM group, the Cohen’s Kappa coefficient for inter-reader agreement was 0.66. The concordance of results between “self-testing” and “rereading” was 99% (103/104). There was one case, in which the participant identified the result as negative, but it was determined as weak positive by a study staff member on rereading. For the inter-operator agreement, the Gwet’s AC coefficient was 0.99. The concordance of results between “self-testing” and “retesting” was 99% (103/104). There was one case in which the participant identified the result as invalid, but it was negative upon retesting by a study staff member.

A total of 21 participants among 105 PWID (20%) and one participant among 104 MSM (1%) received discordant results between self-testing, rereading and retesting. All the discordant results from the 22 participants are listed in Table 6 and classified in four groups. The first group included six participants who incorrectly interpreted their results. All the results of these six participants were read by study staff as positive or weak positive, while after retesting the results of two participants changed from weak positive to negative. Among these six participants, one had poor eyesight, one did not understand the instruction, and two made mistakes during sample collection. The second group included four participants who correctly interpreted their result, but the self-test results were inconsistent with the retesting results performed by study staff. We observed that two of the four participants had incorrect sample collection. Five participants in the third group interpreted their results as invalid which were consistent with rereading by study staff; however, after retesting, three had negative results and all three of these had issues with sample collection. The other two had positive results after retesting but no mistake was observed. The fourth group listed seven participants who were unable to interpret their results even after assistance was provided to four out of seven participants. Four of the tests were read as positive by the study staff and all seven were positive after retesting. Among these seven participants, four had poor eyesight and one made mistakes while collecting the sample.

### 4.4. Acceptability and User Perspectives on HCVST

**Figure 3:**
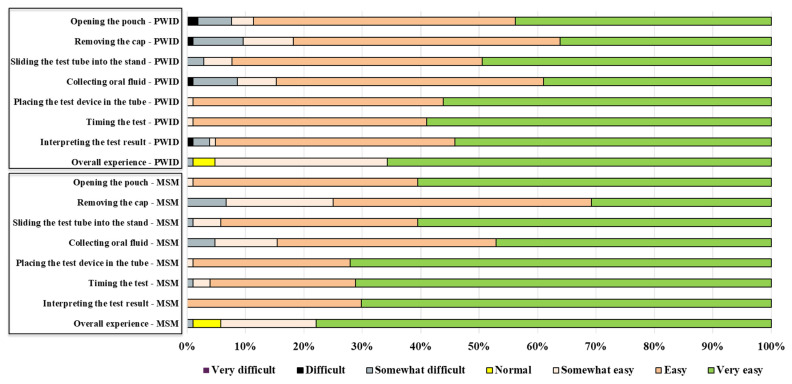
Participants’ perceptions of HCV self-test usability at different steps.

After self-testing, participants provided their perspectives regarding perceptions of HCV self-test difficulty level at different steps (Figure 3).

Table 7 summarizes all the study outcomes. Acceptability was evaluated before and after self-testing. High acceptability in both groups was recorded (>90%). The majority of the participants indicated a preference to test by themselves at home for HCV infection in the future (69.5% in PWID and 76.9% in MSM, *p* = 0.275) and their preferred sample type was oral fluid. Usability in MSM was significantly better than that in PWID, along with higher concordance of results in MSM compared to PWID.

## 5. Discussion

### 5.1. Summary of Findings

To our knowledge, this is one of the first studies to examine the acceptability and usability of HCVST in high-risk populations in a non-clinical setting. The study was conducted with PWID and MSM groups in a province of northern Vietnam. PWID were significantly older (median: 45 vs. 22 years) and had a lower educational level compared to MSM (Table 1).

#### 5.1.1. Usability

The MSM group showed a significantly greater ability to correctly complete the self-testing procedure (66.3% vs. 37.1%, *p* < 0.0001), to complete HCVST without difficulty (71.2% vs. 46.7%, *p* = 0.0004) and without assistance (82.7% vs. 33.3%, *p* < 0.0001) compared to PWID (Table 7). In the PWID group, the collection of oral fluid samples and result interpretation were the two steps at which mistakes were most commonly observed (37.1% and 20%, respectively) (Table 2). These two steps were also the ones most commonly requiring assistance (21.9% and 26.7%). This was attributed to specific factors in these participants, including older age, lower educational background and health factors (poor eyesight, injuries to the hand, or mental condition). The difference in the proportion of participants observed to correctly complete steps, interpret results, and required assistance self-testing indicated that PWID may need more support self-testing, at least in the early days of implementation. Other HIVST studies have also reported that a common mistake in HIV self-testing was sample collection, and older individuals and those with lower levels of education were more prone to make errors. In a systematic review of reliability of the HIV rapid diagnostic test for self-testing [16], it was reported that the most common error that affected test performance was incorrect specimen collection. In another systematic review on acceptability of HIV self-testing, it was reported that accurate performance was associated with the level of education, and misinterpretation of photo results was associated with older age, although generally rare (4.9%) [15].

#### 5.1.2. Concordance of Results

Overall, there was good agreement between inter-reader and inter-operator in both groups. The concordance in the MSM group for both inter-reading and inter-operator (99%) was higher than that in the PWID group (88.6% and 81.9%). With the inter-reading measure, the Cohen’s Kappa coefficient in the PWID group (0.77) was relatively higher than that in MSM group (0.66). For inter-operator measure, the Cohen’s Kappa coefficient in PWID (0.61) was lower than that in MSM group (0.99). The common mistakes we observed that affected the self-test results were incorrect sample collection, errors due to poor eyesight or difficulty in understanding the instruction. Assistance may be needed for those who have issues with reading or understanding the instructions. A demonstration video would be helpful in these cases. In 2019, Kimble et al. evaluated the performance of the OraQuick HCV Rapid Antibody Test in oral fluid when used by 95 patients (48 males and 47 female) for self-testing in the United States [21]. The authors reported that the test kit showed good performance when used by patients: sensitivity and specificity on self-collected oral fluid samples were 88.4%% (95% CI, 74.9–96.1) and 100% (95% CI, 93–100), respectively, when patients interpreted the test results. Sensitivity and specificity were 97.7% (95% CI, 88–99.9) and 98% (95% CI, 89.6–100), respectively, when trained staff interpreted the result. The Cohen’s Kappa coefficient was reported as 0.89 for inter-reading in this study.

#### 5.1.3. Acceptability

There was high acceptability of HCV self-testing with more than 90% agreeing to participate in the study (92.9% of PWID and 98.6% of MSM). After the self-testing experience, the participants expressed a willingness to use an HCV self-test again or to introduce the self-test to family members and friends (Table 7). In 2018, in the first study to report on the acceptability of HCV self-testing performed by un-trained users [22], Guise et al. conducted qualitative rapid assessment to explore acceptability and key challenges of HCV self-testing among 22 study participants across three focus groups of PWID in the UK. The study showed potential acceptability but also revealed multiple concerns associated with self-testing such as poor access to confirmatory testing and care.

### 5.2. The Performance and Applicability of OraQuick HCV Antibody Self-Test

The clinical performance of the OraQuick HCV test has been evaluated and shown to be comparable to that of laboratory-based tests with both serum and oral fluid [23]. Overall, eight studies reported sensitivity and specificity of OraQuick (OraSure Technologies, PA, USA) with a total sample of 9024. The sample size of these studies ranged from 172 to 2183, sensitivities from 90% to 100%, and specificities from 95% to 100% [23]. The pooled sensitivity and specificity were 98% (95% CI: 97%–99%) and 100% (95% CI: 90%–100%), respectively. Among studies that assessed individual oral RDTs, the eight studies showed that OraQuick ADVANCE^®^ had a slightly higher sensitivity (98%, 95% CI: 97–98%) compared to the other oral brands (pooled sensitivity: 88%, 95% CI: 84–92%). The performance characteristics of OraQuick had previously met the quality standards of RDT testing for oral specimen; therefore, in our study, we did not include testing using a reference assay to determine the sensitivity and specificity of the self-testing performed by the un-trained users. We focused mainly on the issues of acceptability and usability of HCV self-testing in two key populations, as these issues are critical in terms of feasibility and scale-up, and to improve the product to be more user-friendly.

Our findings have important implications for further work on self-testing. First, the higher proportion of PWID who experienced difficulties correctly performing the test, interpreting the results independently suggests that some individuals may require assistance, and face-to-face guidance or video demonstration would be useful. Second, we observed several steps where there were consistent difficulties, and we propose specific modifications to the product to improve the usability. The tube containing reagent was designed with a flip top cap, but participants often mistook them for screw cap tubes, which resulted in difficulty in opening the tube. The font size of the written instructions and the photo of result illustration were not large enough for those individuals with poor eyesight. The workflow of the testing procedure could be displayed in vertical order from the upper side down to the lower side in the instruction paper frame to prevent misunderstandings about the correct sequence of steps.

Other issues that need to be addressed are strategies to ensure prompt linkage and access to HCV viral load testing to confirm chronic HCV infection and need for treatment. At present, this represents a major barrier in access to care and treatment, especially among marginalized populations. There are also concerns that self-testing while promoting access to serological testing may result in sub-optimal linkage and uptake of treatment.

The implementation of HCV self-testing in Vietnam is important because key populations account for a significant proportion of the almost one million HCV-infected persons in the country. Based on 2013 estimates, there are around 271,506 PWID, 382,506 MSM and 71,936 sex workers in Vietnam, and all of them should be offered and have regular access to testing for HIV, hepatitis C and B, and sexually transmitted infections [24]. Self-testing represents a potential option to expand this access. Additionally, Vietnam has good experience in implementing HIV self-testing. The national guidelines for community-based HIV testing including HIV self-testing were approved by the Vietnam Ministry of Health in 2018 after one year of piloting in selected provinces [25]. HIV self-testing has now been scaled up country wide. The feasibility and effectiveness of HIV self-testing in case finding and linkages to care has been reported [20]. This represents a strong foundation for the implementation of HCV self-testing once test kits are made available for communities.

This study presented some limitations. The convenience sampling may present a selection bias in PWID and MSM at the selected local sites. Given the low positive rate of anti-HCV in the MSM group, a larger sample size for MSM would be better to assess the concordance of the test results. Also, female sex workers as one of the key populations was not included in this study. Finally, assessment for dual risk and screening for high risk for HCV acute infection were not conducted in this study.

## 6. Conclusions

This study shows a favorable experience with an oral fluid HCV self-test in two key populations, PWID and MSM. Self-testing for HCV may also be a good option for other populations to learn their HCV status. Additional assistance such as demonstration videos or support from health staff or peer educators during the self-testing process may be needed for individuals who conduct self-testing for the first time or who have difficulty in following instructions for users. The study findings provide important information for development of HCVST that may facilitate scale-up of HCV screening and improve coverage of HCV treatment. This emerging evidence provides a strong foundation to support further studies to inform global, regional, and national HCV testing policy and expanded access to HCV testing.

## Figures and Tables

**Table 1:** Baseline demographic characteristics of study participants.

Demographic Characteristics		PWID (*n* = 105)	MSM (*n* = 104)	*p* Value
Age, years, median (range)		45 (31–62)	22 (18–26)	<0.0001
Sex, % (*n*)				
	Male	98.1 (103)	100.0 (104)	0.497
	Female	1.9 (2)	0	
Education, % (*n*)				<0.0001
	Primary school	6.7 (7)	0	
	Secondary school	55.2 (58)	0	
	High school	37.1 (39)	12.5 (13)	
	College	1.0 (1)	87.5 (91)	
Occupation, % (*n*)				<0.0001
	Unemployed	48.6 (51)	2.9 (3)	
	Employed	51.4 (54)	97.1 (101)	
Marital status, % (*n*)				<0.0001
	Married or living with a partner	57.1 (60)	2.9 (3)	
	Divorced, separated or widow	17.1 (18)	1.0 (1)	
	Unmarried	25.7 (27)	96.2 (100)	
Self-reported exposures (ever) to any following risk factors for HCV infection, % (*n*)				
	Injecting non-prescribed drugs	100.0 (105)	0	<0.0001
	Sharing needles	46.7 (49)	0	<0.0001
	Condomless anal intercourse	6.7 (7)	100 (104)	<0.0001
	A surgical procedure	11.4 (12)	15.4 (16)	0.424
	A dental procedure	27.6 (29)	45.2 (47)	0.009
	Sharing shaving tools or toothbrushes	25.7 (27)	28.8 (30)	0.643
	Having a tattoo	32.4 (34)	6.7 (7)	<0.0001
Aware of the existence of self-tests performed at home, % (*n*)		32.4 (34)	87.5 (91)	<0.0001
Previous use of self-test, % (*n*)		1.0 (1)	0	1.000

**Table 2:** Observer assessment of mistakes (using product-specific checklist).

Testing Steps	PWID		MSM		*p* Value
	*n*	%	*n*	%	
Pretesting					
1. Opening the pouch and taking all contents out	0	0	0	0	
2. Reading/using the instructions for use	2	1.9	0	0	0.498
3. Removing the test tube from the pack	0	0	0	0	
4. Removing the cap from the test tube	2	1.9	0	0	0.498
5. Sliding the test tube into the stand	1	1	1	1	1.000
6. Pouring the fluid from the test tube into the stand	4	3.8	1	1	0.369
7. Removing the test device from the pack	0	0	0	0	
Conduct of Test					
8. Touching the test pad	7	6.7	4	3.8	0.538
9. Incorrectly collecting oral fluid sample *	39	37.1	14	13.5	0.0001
10. Wrong placing the test device in the test tube	3	2.9	1	1	0.621
11. Test device coming out of the tube while testing	0	0	0	0	
12. Not reading the result between 20 and 40 min	4	3.8	2	1.9	0.683
Test Interpretation					
13. Wrong interpreting the test result **	21	20	1	1	<0.0001

* Correct manipulation is indicated in the instruction guide provided for participants: “Firmly put the flat pad to the gums and swab along the upper outer gum one time and along lower outer gum onetime”; ** Step considered correct if results read by the study participant agree with rereading by a trained staff.

**Table 3:** Observer assessment of difficulties and steps requiring assistance.

Testing Step, % (*n*)	Difficulty Observed			Assistance Provided		
	PWID	MSM	*p*	PWID *	MSM	*p*
	*n* = 105	*n* = 104		*n* = 105	*n* = 104	
Opening the pouch and taking all the contents out	6.7 (7)	4.8 (5)	0.768	15.2 (16)	2.9 (3)	0.003
Reading/using the instructions for use	NA	NA		16.2 (17)	0 (0)	<0.0001
Removing the cap from the test tube	21.0 (22)	19.2 (20)	0.863	21.9 (23)	1 (1)	<0.0001
Sliding the test tube into the stand	11.4 (12)	1.9 (2)	0.01	17.1 (18)	0 (0)	
Collecting the oral fluid sample	NA	NA		21.9 (23)	0 (0)	<0.0001
Placing the test device in the test tube	1.0 (1)	1.9 (2)	0.621	15.2 (16)	1.9 (2)	0.0008
Interpreting the test result	13.3 (14)	1.9 (2)	0.003	26.7 (28)	2.9 (3)	<0.0001

* Three participants had previous problems with their hand/s (injury and disability) and eight participants had poor eyesight that prompted request for assistance from study staff.

**Table 4:** Association between personal factors and usability of the hepatitis C virus (HCV) self-test.

Demographic Characteristics	Mistake			Difficulty			Assistance		
	*n*	%	*p*	*n*	%	*p*	*n*	%	*p*
Age, years, median (range)									
<22 (N = 51)	21	41.2	0.0012	15	29.4	0.0030	9	17.6	<0.0001
22–45 (N = 107)	44	41.1		40	37.4		41	38.3	
>45 (N = 51)	36	70.6		31	60.8		38	74.5	
Sex, % (*n*)									
Male (N = 207)	48.8	101		41.1	85		42.0	87	
Female (N = 2)	0.0	0		50.0	1		50.0	1	
Educational level, % (*n*)									
Primary school (N = 7)	4	57.1	0.0001	5	71.4	0.0010	6	85.7	<0.0001
Intermediate school (N = 58)	41	70.7		33	56.9		43	74.1	
Secondary school (N = 62)	27	43.5		23	37.1		22	35.5	
College (N = 92)	29	31.5		25	27.2		17	18.5	
Occupation, % (*n*)									
Unemployed (N = 54)	32	59.3	0.0875	25	46.3	0.4641	29	53.7	0.0651
Employed (N = 155)	69	44.5		61	39.4		59	38.1	
Marital status, % (*n*)									
Married or living with a partner (N = 63)	46	73.0	<0.0001	38	60.3	0.0011	39	61.9	<0.0001
Divorced, separated or widow (N = 19)	7	36.8		6	31.6		12	63.2	
Unmarried (N = 127)	48	37.8		42	33.1		37	29.1	

**Table 5:** The concordance of HCV testing results performed by the participants (self-testing), re-interpreted by the health care staff (rereading) and performed by the study staff (retesting) in PWID and MSM.

**PWID (*n*)**		**Rereading by Trained Staff** **(Inter-Reader)**				**Retesting by Trained Staff** **(Inter-Operator)**			
		**Positive**	**Negative**	**Invalid**	**Unsure**	**Positive**	**Negative**	**Invalid**	**Unsure**
Self-Testing	Positive	64	0	0	0	64	0	0	0
	Negative	5	25	0	0	8	22	0	0
	Invalid	0	0	4	0	2	2	0	0
	Unsure	4	0	3	0	7	0	0	0
Concordance (%)		Between self-testing and rereading: 88.6%				Between self-testing and retesting: 81.9%			
Cohen’s Kappa		0.77				0.61			
**MSM (*n*)**		**Rereading by Trained Staff** **(Inter-Reader)**				**Retesting by Trained Staff** **(Inter-Operator)**			
		**Positive**	**Negative**	**Invalid**	**Unsure**	**Positive**	**Negative**	**Invalid**	**Unsure**
Self-Testing	Positive	0	0	0	0	0	0	0	0
	Negative	1	102	0	0	0	103	0	0
	Invalid	0	0	1	0	0	1	0	0
	Unsure	0	0	0	0	0	0	0	0
Concordance (%)		Between self-testing and rereading: 99%				Between self-testing and retesting: 99%			
Cohen’s Kappa		0.66				0.99 *			

* Gwet’s AC coefficient.

**Table 6:** Discordant results among self-testing, rereading (interpreted by study staff) and retesting (by the study staff) in PWID and MSM groups.

Study ID	Self-Testing	Rereading	Retesting	Touching the Pad	Collecting Oral Fluid Sample	Assistance to Interpret Result	Observations for Discordant Results
**Wrong Result Interpretation by Participants (*n* = 6)**							
PWID-011	Negative	Positive	Positive	No	Correct	Yes	Participant did not understand the instruction
PWID-022	Negative	Positive	Positive	No	Correct	No	
PWID-048	Negative	Weak Positive	Positive	No	Correct	Yes	Participant had poor eyesight
PWID-082	Negative	Positive	Positive	No	Incorrect	Yes	Participant placed device on the tongue and swabbed the teeth
PWID-097	Negative	Weak positive	Negative	No	Incorrect	No	Participant placed device on the tongue
MSM-062	Negative	Weak Positive	Negative	No	Correct	Yes	
**Correct Result Interpretation by Participant but Retesting Result Differing from Self-Testing Result (*n* = 4)**							
PWID-073	Negative	Negative	Positive	No	Correct	No	
PWID-075	Negative	Negative	Positive	No	Incorrect	No	Participant swabbed the teeth
PWID-105	Negative	Negative	Weak positive	No	Correct	No	
PWID-106	Negative	Negative	Positive	No	Incorrect	No	Participant swabbed tongue and teeth
**Correct Interpretation of Invalid Results in Self-Testing (*n* = 5)**							
PWID-010	Invalid	Invalid	Positive	No	Correct	No	
PWID-077	Invalid	Invalid	Positive	No	Correct	No	
PWID-017	Invalid	Invalid	Negative	No	Incorrect	No	Participant swabbed on one gum only
PWID-054	Invalid	Invalid	Negative	No	Incorrect	No	Participant did not use the whole pad
MSM-066	Invalid	Invalid	Negative	Yes	Incorrect	No	Participant did not swab firmly enough
**Unsure How to Interpret the Results in Self-Testing (*n* = 7)**							
PWID-036	Unsure	Positive	Positive	No	Correct	Yes	Participant had poor eyesight
PWID-052	Unsure	Positive	Positive	No	Correct	Yes	Participant had poor eyesight
PWID-053	Unsure	Positive	Positive	No	Correct	Yes	
PWID-092	Unsure	Positive	Positive	No	Incorrect	No	Participant did not use the whole pad
PWID-043	Unsure	Invalid	Positive	No	Correct	Yes	
PWID-059	Unsure	Invalid	Positive	No	Correct	No	Participant had poor eyesight
PWID-064	Unsure	Invalid	Positive	No	Correct	No	

**Table 7:** Summary of acceptability, preference, usability and concordance results of HCV self-testing (HCVST).

% (*n*)		PWID	MSM	*p* Value
Acceptability, % (*n*)		*n* = 105	*n* = 104	
Before self-testing				
	The proportion of participants among eligible subjects who agreed to participate and perform HCV self-testing	92.9 (131/141)	98.6 (139/141)	0.034
	Ready to use HCV self-test if available	98.1 (103)	100.0 (104)	0.498
After self-testing				
	Willing to use HCV test again	91.4 (96)	98.1 (102)	0.058
	Willing to recommend the test to family and friends	99 (104)	97.1 (101)	0.369
	Taking the tests to family member/friend	99.0 (104)	97.1 (101)	0.503
Preferences on HCVST, % (*n*)		*n* = 105	*n* = 104	
Preferred approach to test for HCV in the future				
	By myself at home	69.5 (73)	76.9 (80)	0.275
	By myself at a health center	7.6 (8)	10.6 (11)	0.481
	In a community center by a healthcare worker	8.6 (9)	5.8 (6)	0.593
	In a screening campaign	0 (0)	2.9 (3)	0.121
Preferred sample type				
	Prefer oral fluid-based test	79 (83)	67.3 (70)	0.416
	Prefer blood-based test	10.5 (11)	26.9 (28)	
	No preference	10.5 (11)	5.8 (6)	
Steps to take if results of self-test reactive				
	Contact healthcare facility	95.2 (100)	83.7 (87)	0.006
	Contact pharmacy	1.9 (2)	1.9 (2)	1.000
	Do a confirmatory test	28.6 (30)	57.7 (60)	<0.0001
	Seek advice from a family member/community	13.3 (14)	11.5 (12)	0.831
	Do not know	1.0 (1)	0 (0)	1.000
Knowledge about HCV treatment				
	Know that HCV can be cured	64.8 (68)	55.8 (58)	0.205
	Know that there is a treatment but not sure about the cure	10.5 (11)	19.2 (20)	0.083
	Not sure if there is treatment	4.8 (5)	8.7 (9)	0.284
	There is no treatment or cure	0 (0)	1.9 (2)	0.246
Usability, % (*n*)				
	Correctly completing self-testing without any mistake	37.1 (39)	66.3 (69)	<0.0001
	Correctly collecting oral fluid	62.9 (66)	86.5 (90)	0.0001
	Correctly interpreting the self-test results	80 (84)	99 (103)	<0.0001
	Completing self-test procedure without difficulty	46.7 (49)	71.2 (74)	0.0004
	Completing self-test procedure without assistance	33.3 (35)	82.7 (86)	<0.0001
Concordance of Results				
Inter-reader agreement				
	Concordance, % (*n*)	88.6% (93)	99% (103)	0.005
	Kappa value	0.77	0.66	
Inter-operator agreement				
	Concordance, % (*n*)	81.9% (86)	99% (103)	<0.0001
	Kappa value	0.61	0.99	

## Data Availability

The data presented in this study are available on request from the corresponding author. The data are not publicly available due to confidentiality.
